# Effectiveness of the internet-based Unified Protocol transdiagnostic intervention for the treatment of depression, anxiety and related disorders in a primary care setting: a randomized controlled trial

**DOI:** 10.1186/s13063-022-06551-y

**Published:** 2022-08-31

**Authors:** Ladislav Timulak, Derek Richards, Louise Bhandal-Griffin, Patrick Healy, Juliana Azevedo, Graham Connon, Elaine Martin, Aoife Kearney, Conor O’Kelly, Angel Enrique, Nora Eilert, Sorcha O’Brien, Siobhan Harty, Alberto González-Robles, Elizabeth H. Eustis, David H. Barlow, Todd J. Farchione

**Affiliations:** 1 Trinity College Dublin, Dublin, Ireland; 2 SilverCloud Science, SilverCloud Health, Dublin, Ireland; 3 Health Service Executive, Dublin, Ireland; 4 University of Zaragoza, Zaragoza, Spain; 5 Boston University, Boston, USA

## Abstract

**Background:**

Research has shown that internet-based cognitive behavioural therapy (iCBT) can be a very promising solution to increase access to and the dissemination of evidence-based treatments to all of the population in need. However, iCBT is still underutilized in clinical contexts, such as primary care. In order to achieve the effective implementation of these protocols, more studies in ecological settings are needed. The Unified Protocol (UP) is a transdiagnostic CBT protocol for the treatment of emotional disorders, which includes depression, anxiety and related disorders, that has shown its efficacy across different contexts and populations. An internet-based UP (iUP) programme has recently been developed as an emerging internet-based treatment for emotional disorders. However, the internet-delivered version of the UP (iUP) has not yet been examined empirically. The current project seeks to analyse the effectiveness of the iUP as a treatment for depression, anxiety and related emotional disorders in a primary care public health setting.

**Methods:**

The current study will employ a parallel-group, randomized controlled trial design. Participants will be randomly assigned to (a) the internet-based Unified Protocol (iUP), or (b) enhanced waiting list control (eWLC). Randomization will follow a 2:1 allocation ratio, with sample size calculations suggesting a required sample of 120 (iUP=80; eWLC=40). The Mini-International Neuropsychiatric Interview (M.I.N.I.) will be used for assessing potential participants. The Overall Anxiety Severity and Impairment Scale (OASIS) and the Overall Depression Severity and Impairment Scale (ODSIS) as well as other standardized questionnaires will be used for assessments at baseline, 4 weeks, 8 weeks and 12 weeks from baseline and for the iUP condition during the follow-up.

**Discussion:**

Combining the advantages of a transdiagnostic treatment with an online delivery format may have the potential to significantly lower the burden of emotional disorders in public health primary care setting. Anxiety and depression, often comorbid, are the most prevalent psychological disorders in primary care. Because the iUP allows for the treatment of different disorders and comorbidity, this treatment could represent an adequate choice for patients that demand mental health care in a primary care setting.

**Trial registration:**

ISRCTN18056450 10.1186/ISRCTN18056450.

## Introduction

Epidemiological studies have found that anxiety (e.g. social anxiety, generalized anxiety, panic disorder), anxiety-related disorders (e.g. posttraumatic stress disorder, obsessive-compulsive disorder) and unipolar mood disorders (e.g. depression), sometimes referred to as emotional disorders (ED) [1], account for the highest prevalence of psychological disorders on a global scale [2]. There is a significantly high lifetime prevalence rate for depression, anxiety and anxiety-related disorders coupled with very high comorbidity rates [3]. In Ireland, depression and anxiety are the most common psychological conditions with which patients present to their General Practitioner (GP) [4] and are the most common referrals to primary care mental health settings [5]. These disorders are also associated with enormous costs and disability both directly and indirectly [6]. In relation to treatment methods, research has indicated that psychological treatment is equally as effective as pharmacological [7], and according to McHugh and colleagues [8], clients demonstrated a preference for psychological interventions over pharmacological. Effective evidence-based psychological treatments have been developed and refined; however, many people do not have the resources to access these [9]. The provision of psychological interventions in the context of public health, in some countries like Ireland, is also impacted by the sheer volume of referrals that require the use of extensive wait-lists [10]. In the past 20 years, the emergence of internet-based treatments started to address these gaps in dissemination. This is particularly the case in countries that use low-intensity interventions and a stepped-care approach as part of their public health provision, e.g. [11].

Internet-based cognitive behavioural therapy (iCBT) was developed to improve access to treatment of disorders such as depression or anxiety while maintaining treatment efficacy as well as cost-effectiveness [12]. A growing body of research has shown the efficacy and effectiveness of iCBT for anxiety and depression, e.g. [13–15]. Compared to face-to-face treatments, online treatments offer important advantages not only in terms of dissemination and access to evidence-based treatments, and alleviating personal barriers such as the stigma of physically attending a service, but also by decreasing the workload of mental health care providers with a significant reduction in costs [12].

Research over the past 30 years has provided a myriad of disorder-specific CBT protocols for depression and anxiety [16]. However, although effective, disorder-specific CBT has important shortcomings with regard to dissemination and effective uptake, such as their higher costs (e.g. in terms of training) [17] and less efficiency in addressing comorbid presentations [18]. These reasons, along with the literature showing the role of common mechanisms underlying emotional disorders, shared aetiology and shared comorbidity, have triggered the development and study of transdiagnostic treatments [19, 20]. The Unified Protocol (UP) is a transdiagnostic CBT protocol that addresses the common psychopathological processes underlying emotional disorders, with a particular focus on neuroticism and emotion dysregulation, aspects that play a key role in the onset and maintenance of these disorders [19, 20]. The UP has shown efficacy in a face-to-face format, that it is as effective as well-established disorder-specific CBT [21, 22]. The recent meta-analyses showed its efficacy for depression, anxiety and related conditions [23, 24].

In terms of the number of studies conducted, the UP is a true leader among transdiagnostic interventions [23–25]. However, an internet-delivered version of the UP (iUP) for adults, with the exception of a Spanish UP based programme that also included other elements [26], a German [27] and a Romanian version [28] has not yet been examined. These three previous interventions showed promising results in terms of their outcomes and retention. The three non-English interventions, unlike the current study, were not used in a routine public health primary care setting that is characterized by waiting lists which may require easily disseminated interventions that can reach clients while they are on the wait-list. The current provision in the context of the Irish public health service typically offers minimal self-directed low-intensity interventions. The current study is the first online adaptation of the UP developed by the original developers of the UP and a leading digital mental health company, SilverCloud Health. The current study is also an initial test of the efficacy of the iUP in the context of the setting, primary care, characterized by a high prevalence of (comorbid) depression and anxiety and related disorders.

### Aims of the current research

Our main aim is to test the initial effectiveness of the iUP for depression, anxiety and related emotional disorders against an enhanced wait-list control (eWLC; enhanced in terms that the service routinely offers a number of resources for the clients waiting for an intervention—see below) in the Irish public health primary care service (the Health Service Executive [HSE]). This project aims to answer the following principal research questions:

1. Will the iUP intervention be significantly more effective than the enhanced waiting list control group in treating depression, anxiety and related disorders among the clients in the public primary care?
2. What are the reported experiences (helpful and unhelpful aspects of the treatment and the treatment’s impact) of the clients undergoing iUP as their treatment?

## Method

### Design

The current study will employ a parallel-group, exploratory randomized controlled trial design. The study will follow the CONSORT statement [29], CONSORT E-Health guidelines [30] and the SPIRIT guidelines (Standard Protocol Items: Recommendations for Interventional Trials) [31]. Participants will be randomly assigned to (a) the internet-based Unified Protocol (iUP), or (b) enhanced waiting list control (eWLC). Randomization will follow a 2:1 allocation ratio, with sample size calculations suggesting a required sample of 120 (iUP=80; eWLC=40). Assessments will take place at baseline, 4 weeks, 8 weeks and 12 weeks from baseline. For ethical reasons, the eWLC group participants will be offered iUP treatment after 12 weeks. iUP group participants will be followed up at 16 weeks, 20 weeks and 24 weeks from baseline, so that we can examine (short-term) stability of any effects achieved.

### Participants and study setting

Participants will be adults (18 years old or older) with anxiety and/or depressive symptoms that attend primary care services in Ireland to seek mental health care. To be eligible, participants will have to have a score of ≥ 8 on the Overall Anxiety and Severity Impairment Scale (OASIS) [32] and/or a score of ≥8 on the Overall Depression Severity and Impairment Scale (ODSIS) [33]. The study will take place in a naturalistic setting within a Dublin-based primary care psychology service which is part of the Health Service Executive (HSE) in Ireland. Referrals are made via General Practitioners (GP), allied services, or self-referral. Average wait-list times can range from 3 to 12 months. At the end of the wait-list period, the service usually offers an initial intake assessment followed by an individual care plan, including individual and/or group psychotherapy, delivered by psychologists trained in a variety of therapeutic modalities. The service offers some support for clients on the wait-list (including a lecture series on stress control, a list of self-help books and the possibility to have a 30-min consultation with a psychologist in an advice clinic).

### Sample size

Sample size was determined in relation to the planned analysis of primary outcomes (i.e. linear mixed models of anxiety/depression severity) and calculated in the R package ‘powerlmm’ [34]. Model parameters (random intercept/slope, residual variance) required for this calculation were estimated from similar data from a previous trial on a transdiagnostic intervention in primary care [26]. Given a 2:1 randomization ratio, longitudinal data collected at four timepoints across both groups, 30% research attrition at the primary endpoint and a moderate post-treatment between-group effect size of *d*=0.5 in line with previous research [21, 35], a sample size of 120 (iUP=80, WLC=40) was estimated to yield sufficient statistical power of 0.83 (a Bonferroni-adjusted *p* value of 0.025 to account for two primary outcome measure would reduce power to 0.75; at a lower attrition rate of 20% rather than 30%, power of 0.80 would be reached even with the Bonferroni-adjusted *p* value).

#### Intervention

##### Internet-based Unified Protocol (iUP)

The iUP is an eight-module online intervention that is based on the second edition of the UP [21, 36]. It includes the five core CBT modules in the UP that target neuroticism (i.e. negative affect and aversive reactivity to emotions) and subsequent emotion motivated avoidant coping [37]. See Table 1 for an overview of the modules. The programme is self-directed, so the client can proceed through the programme at their own pace. The recommended time to spend on a module is initially 1 week, but starting with module 3, and for the rest of the core modules in the UP (Table 1), the supporter (see below) may suggest extra time, 2 or more weeks. Supporters particularly recommend that participants spend 2–4 weeks on module 7 (Emotion Exposures). Specific recommendations from supporters will also consider participant pace, progress and engagement with the programme (e.g. if someone does not complete a specific module 1 week, they may be encouraged to complete it the following week). This intervention will be delivered on the SilverCloud platform. SilverCloud Health is a global leader in the development of computerized psychological interventions for depression, anxiety, stress and comorbid long-term conditions. The intervention will be delivered on a Web 2.0 platform using media-rich interactive content. Consistent with other SilverCloud programmes, each module follows a structured format and includes videos, a psychoeducational content, interactive activities and tools, instructions for practicing skills and summaries of the modules. Clients’ progress and use of the iUP will be reviewed by a supporter, who will provide feedback on the client’s use of the programme. Their support will consist of email messages to the clients that provide encouragement and suggestions for the use of the programme. The feedback will be provided every 6 to 10 days. The supporters will be assistant psychologists and/or trainee psychologists. They will be under supervision of a qualified psychologist. All participating clinicians will be trained by an UP trainer in the delivery of support for iUP and in addition will receive training on the platform and how to deliver supporter reviews to participants.

**Table 1:** Modules in the Internet-based UP

Module name	Brief description
Getting Started	This module introduces the UP and how it can be helpful in managing emotions. Additional content focuses on goal setting and motivation. Users set specific goals and consider the pros and cons of changing.
Understanding Your Emotions	This module provides psychoeducation about the nature and function of emotions. Users learn that all emotions can be broken down into 3 components: thoughts, physical sensations and behaviours, that each interact with the others. Antecedents or triggers of emotions are identified, as well as short- and long-term consequences of responses to emotions.
Mindful Emotion Awareness^a^	This module introduces mindful emotion awareness as a skill to use in response to emotion. The two components are present-focused awareness and non-judgmental awareness. Several ways to practice this skill are covered including a guided meditation exercise, practicing while watching an emotional video clip, and applying the skill in daily life.
Flexible Thinking^a^	This module focuses on thoughts and their connection to emotions. Users learn how to identify negative thinking patterns and develop skills to practice thinking more flexibly.
Emotional Behaviours^a^	This module focuses on the behavioural component of emotions. Users learn how identify emotional behaviours (behaviours to avoid or decrease emotions) and how to practice changing their behaviour through alternative actions.
Facing Physical Sensations^a^	This module focuses on the physical sensations component of emotions. Users learn about the importance of context and their interpretation of physical sensations, and complete interoceptive exposures exercises to physical sensations (e.g. hyperventilation, straw breathing, spinning and running in place).
Emotion Exposures^a^	This module focuses on emotion exposures, activities, or situations designed to intentionally bring up strong emotions. Users create a hierarchy of exposures, and learn how to practice applying all of the skills they have already learned in the programme while preparing for, engaging in, and debriefing from exposures.
Progress and Practice	This final module focuses on reviewing progress, relapse prevention, and making a plan to continue practicing skills after the programme.

^a^ indicates core modules in the UP

##### Enhanced Wait-List Control (eWLC)

The participants placed on the wait-list may avail of several resources suggested to them. These include lecture series on stress control, list of self-help books and the possibility to have a 30-min consultation with a psychologist in an advice clinic. The uptake of those resources is self-directed. The record will be taken of what resources, if any, participants actually availed of.

### Eligibility criteria

Clients will be selected based on the following inclusion/exclusion criteria. Preliminary screening of the existing referrals will include potential participants that will be of minimum age of 18 years old, fluent in English, have access to the Internet and an email account and have symptoms of anxiety and/or depression referenced as primary presenting problem(s) on the referral form from the General Practitioner (GP). The full inclusion criteria will then include a score of ≥ 8 on OASIS [32, 38] and/or a score of ≥ 8 on ODSIS [33]. Exclusion criteria will include an increased risk of suicidality intent or ideation (at least moderate scores on the M.I.N.I [39]. and/or a score > 2 on item 9 from the PHQ-9 [40], psychotic disorder, manic or hypomanic episode, bipolar disorder, eating disorder, anti-social personality disorder and/or alcohol/substance use disorder as indicated by the M.I.N.I., references to cognitive impairment on the referral form, or a receipt of another psychological treatment at the start of the study. It is envisaged that pharmacological treatment during the treatment period will be allowed provided that it was stabilized 6 weeks prior the start of being involved in the study. Any changes to it will be monitored afterwards.

### Recruitment procedures

#### Participant recruitment

**Fig. 1:**
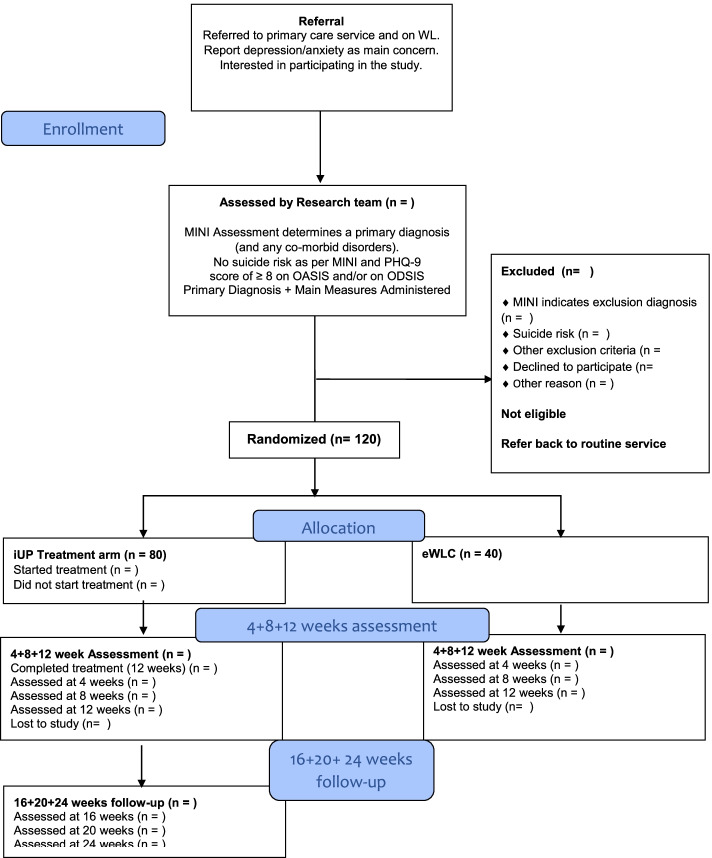
CONSORT flow chart

Recruitment has begun in November 2021 and will run until the required sample size is reached. This is estimated to take between 18 and 24 months. Potential participants will be selected in two ways: from the routine primary care service’s referral/waiting list. An initial telephone screening interview will be conducted by a clinician (psychologist, trainee psychologist or assistant psychologist) working on the research team to determine if the individual meets the preliminary eligibility criteria. If eligibility criteria are met, the individual will be invited to participate in the study. An overview of the study will be provided, and the individual will be given the opportunity to ask questions about the study. Those who wish to participate will be emailed a link to the study’s website, through which they will be asked to provide informed consent digitally. Participants will be told that they may withdraw from the study at any time. Once consent is obtained, a follow-up structured interview will be scheduled, where a clinician (psychologist, trainee psychologist or assistant psychologist) will administer the Mini-International Neuropsychiatric Interview (M.I.N.I.) as well as other standardized questionnaires. Clinicians administering the M.I.N.I. will undergo a specific training programme prior to using the tool to ensure the quality standard is maintained. The M.I.N.I. evaluation will allow the clinician to assign participants into diagnostic categories. Following the recruitment procedure, participants will be randomly allocated into the treatment or eWLC group using a ratio of 2:1. Participants will then be advised to which group they have been assigned (for the flow of participants, see the CONSORT Fig. 1). Participants will be instructed on next steps for their treatment group.

#### Clinician recruitment

All participating clinicians/supporters (assistant psychologists and trainees psychologists who will be offering asynchronous support for clients utilizing iUP (Supporters) and psychologists who will be offering supervision of assistant/trainee psychologists) from the primary care site will be briefed on relevant aspects of study design and objectives. They will participate as both clinical professionals supporting treatment and will also be involved in the research itself.

### Risk management

The iUP programme is not intended to provide crisis support or intervention to individuals who are at risk of suicide. Risk will be assessed, monitored and managed as part of an ongoing duty of care to all participants in the study and this will correspond with the site’s own governance and risk procedures. The M.I.N.I. and PHQ-9 measure will be used to provide an initial risk assessment whereby individuals who score > 2 on the item relating to suicide and self-harm will be excluded from the study and referred to get additional support. The score on PHQ-9 will also be reviewed at weeks 4, 8, 12 and also at weeks 16, 20 and 24 and in case of elevated risk (score > 2 on the item relating to suicide and self-harm), further follow-up questions will be administered (Do you have any current plans to end your life? Have you made any current preparations toward ending your life? How likely is it that you will act on these thoughts or plans to end your life?) and this will be brought to the attention of clinical supervisor who will determine the next steps (e.g. follow-up with the client, referral). Scores on measures will be reviewed at the end of treatment to determine whether the participant can be discharged or will be referred for further treatment within the service. Any further referrals will be recorded.

### Randomization

Following the initial assessment and providing that the participant meets criteria for being included in the study, participants will be randomly allocated at an individual level using an algorithm developed by a computer scientist [41] and executed independently of the research team, employing random permuted blocks using block sizes of 6, and including stratification within a 2:1 allocation ratio between treatment and waiting list control groups. Post assessment, the clinician/researcher administering the M.I.N.I. opens concealed allocation and will then inform the participant of their allocation into either the wait-list or treatment group. The clinicians/supporters carried out the support of the clients in the intervention could not be blinded to allocation for practical reasons; however, the clinicians/researchers conducting the pre-trial assessment are going to be blind to the condition allocation during the assessment.

## Measures

### Diagnostic measure

#### The Mini-International Neuropsychiatric Interview (M.I.N.I. [39])

The M.I.N.I. is a brief structured psychiatric interview, which was designed as a diagnostic tool to identify the most common diagnosis in the DSM-5 [42] and ICD-10 [43]. The M.I.N.I. can be administered in as little as 15 min (mean 18.7 ± 11.6 min, median 15 min) and has similar reliability and validity to similar longer instruments. Clinicians/researchers administering the M.I.N.I will undergo a specific training programme prior to using the tool to ensure the quality standard is maintained. All clinicians administering the M.I.N.I will receive a training in the use of this tool.

### Primary outcomes measures

#### The Overall Anxiety Severity And Impairment Scale (OASIS, [32])

The OASIS is a 5-item measure that can be used to assess the impairment and severity which is associated with multiple anxiety disorders. Items are rated from 0 to 4 and summed to determine the overall score. A score of 8 or above is indicative of a probable anxiety disorder. The OASIS shows good psychometric qualities [38].

#### The Overall Depression Severity and Impairment Scale ODSIS (ODSIS***;*** [33])

The ODSIS is a 5-item self-report measure that can be used to assess severity and impairment with any depressive symptoms. Items are rated from 0 to 4 and summed to determine the overall score. A score of 8 or above is indicative of a probable depressive disorder. The ODSIS shows good psychometric qualities [33].

### Secondary outcome measures

#### Work and Social Adjustment Scale (WSAS; [44])

The WSAS is a 5-item scale which assesses functional impairment in work and social areas. It is a self-report measure that has been utilized with patients with depression and anxiety in primary care contexts.

#### Generalized Anxiety Disorder-7 (GAD-7; [45])

The GAD-7 is based on the DSM-IV diagnostic criteria for GAD and measures both the symptoms and severity of anxiety. GAD-7 displays good internal validity and good convergent validity with other anxiety scales. GAD-7 is a 7-item self-report instrument routinely used in the Irish and UK public health primary care with a cut-off score 8 and higher [46].

#### Patient Health Questionnaire-9 (PHQ-9; [40])

The PHQ-9 is a 9-item self-report scale with sound psychometric qualities measuring the severity of depression. The measure has the cut-off total score ≥ 10 [46]. PHQ-9 is routinely used in the Irish and UK public health primary case and therefore will be used for all clients in this study too.

### Disorder-specific measures

In addition to primary and secondary measures, there will be additional disorder-specific measures applied to the client depending on their primary diagnosis according to M.I.N.I. For the purpose of the primary diagnosis of depression, PHQ-9 will be used. In case of generalized anxiety disorder, it will be GAD-7. The scales listed here will be applicable to other primary diagnoses such as panic disorder, social anxiety, OCD and PTSD.

#### Panic Disorder Severity Scale-Self Report (PDSS-SR; [47])

The PDSS-SR is a 7-item measure to assess both frequency and severity of panic disorder symptoms. The seven items are rated on a 5-point scale that ranges from 0 to 4. The PDSS-SR assesses impairment in functioning; avoidance of situations and physical sensations; and frequency of panic, distress during panic and panic-focused anticipatory anxiety. A score of 9 or above is indicative of caseness. The scale has sound psychometric qualities.

#### Social Phobia Inventory (SPIN; [48])

The SPIN is a 17-item self-report scale that assesses symptoms of social anxiety disorder (each item scores 0 to 4). SPIN assesses the domains of social anxiety and asks users to reflect on their experiences over the past week. The scores are totalled to gain a representation of symptom severity with the score 19 and above meeting the clinical caseness. The scale has sound psychometric qualities.

#### Obsessive-compulsive Inventory-Revised (OCI-R; [49])

The OCI-R is an 18-item self-report scale that assesses obsessive-compulsive disorder symptoms, each item is rated on a 5-point Likert scale (0–4) with the cut-off score being 21 and above. The scale shows good psychometric qualities.

#### PCL-5 ( [50])

This is a 20-item self-report measure assessing symptoms of posttraumatic stress disorder (PTSD) according to DSM-5. Each symptom is scored on a scale 0–4 with the score 31 and above being a clinical cut-off. It has appropriate psychometric qualities.

#### Client Change Interview Protocol (CCIP; [51])

A written questionnaire variant of CCIP will be used to obtain clients’ answers to open ended questions post-intervention such as: What changes, if any, have you noticed in yourself since the internet-based Unified Protocol intervention started? Has anything changed for the worse for you since the internet-based Unified Protocol intervention started? Is there anything that you wanted to change that has not since the internet-based Unified Protocol intervention started? Can you sum up what has been helpful about your internet-based Unified Protocol intervention? What kinds of things about the intervention have been hindering, unhelpful, negative or disappointing for you? Were there things in the intervention which were difficult or painful but still OK or perhaps helpful? What were they? Has anything been missing from your treatment?

#### Engagement and usage measures

Online metrics of use will allow the study to measure and report on participants’ adherence to treatment (use) versus non-adherence to treatment (non-use), which is recommended by the CONSORT E-HEALTH guidelines [30]. A summary of all measures and time points is in Table 2.

**Table 2:** A description (SPIRIT diagram) of enrolment, intervention and assessment

	Study period							
	Enrolment	Allocation	Post-allocation					
Timepoint	***-t***_***1***_	0	***Both conditions***			***Follow-up iUP only***		
			Week 4	Week 8	Week 12	Week 16	Week 20	Week 24
**Enrolment:**								
**Eligibility screening**	X							
**Informed consent**	X							
**Baseline assessment**	X							
**Study suitability**	X							
**Allocation**		X						
**Interventions:**								
***iUP***			**x**	x	x			
***eWLC***						x	x	x
**Assessments:**								
***OASIS***	X		X	X	X	X	X	X
***ODSIS***	X		X	X	X	X	X	X
***M.I.N.I.***	X							
***PHQ-9***	X		x	x	x	x	X	X
***GAD-7***	X		X	X	X	X	X	X
***WSAS***	X		X	X	X	X	X	X
***Engagement and usage***	X							
***Primary diagnosis-specific measure***	X		x	x	X	X	X	X
***CCIP***					X			

SPIRIT diagram of enrolment, intervention and assessment. Legend: *CCIP* Client Change Interview Protocol, OASIS Overall Anxiety Severity and Impairment Scale, *ODSIS* Overall Depression Severity and Impairment Scale, *PHQ-9* Patient Health Questionnaire, *GAD-7* Generalized Disorder 7, *WSAS* Work and Social Adjustment Scale, *M.I.N.I.* Mini-International Neuropsychiatric Interview

### Data analysis

All the analyses will follow the intention-to-treat principle and reporting of the results will adhere to CONSORT recommendations. Missing data analysis will explore missing data patterns and mechanisms. Multiple imputation will be considered where deemed necessary. Linear mixed models will be used to evaluate primary and secondary outcomes. In these models, intercepts and/or slopes will be allowed to vary on the level of the individual where appropriate and time and treatment group as well as their interaction will be included as fixed effects. To allow for the nesting and comparing of models in terms of model fit, models will be estimated via maximum likelihood estimation during model building. Final models will utilize restricted maximum likelihood estimation. Separate linear mixed models utilizing only data from the iUP group will be built to assess the maintenance of effects into follow-up, including random intercepts/slopes and time as a fixed effect. Between-group Cohen’s *d* effect sizes will be calculated based on raw standard deviations [52]. The qualitative data will be analysed using a descriptive-interpretive approach [53].

### Data management

All potential participants for the project will be given a referral code. Participants who proceed from assessment to the trial will be given a trial code. The pre-therapy assessment interview including a shortened version of the M.I.N.I. will take place over the phone. All other screening and assessment information will be gathered via the Qualtrics platform. Data will be collected in a pseudo-anonymized way (not immediately identifiable when looking at the data set but by using codes the data may be linked back to the individual if required); however, once all data is collected, it will be transferred to a database via Qualtrics and completely anonymized for each participant.

All regulations set by the ethics committees as well as data protection recommendation offered by the Data Protection Impact Assessment will be observed. The integrity of the main analyses will be secured by parallel datasets with regularly updated versions with one of them being in anonymized form. Data management procedures will be shared with the Trial Management Group and Trial Steering Committee (see below).

### Governance and oversight of the trial

The Trial Management Group (TMG) will be established to oversee day-to-day operations of running the trial such as training of supporters, recruitment of participants, clinical governance and ethical issues, adverse events and data management processes. The TMG will meet quarterly or as needed. It will consist of the principal investigator (PI), co-principal investigator, trial managers and service representatives. The TGM will regularly discuss any potential changes to the trial protocol that will then be brought to the Trial Steering Committee (TSC). The TSC will consist of the abovementioned members of the TMG, an independent academic, a public health service manager responsible for the services in the area and a service user representative. The TSC will meet every 6 months over the course of the project and will have the function of overseeing the project. The PI will report to the committee on progress regarding the trial and seek perspectives from the TSC regarding any issues arising.

## Discussion

This project aims to make contributions to the existing state of the art of our knowledge about the efficacy of the Unified Protocol (UP) in its varied forms of delivery ( [24]). This is the first study to analyse the effectiveness of an online version of the UP (iUP) in primary care in an English-speaking country. It will also be the first online version of the UP tested that is being developed in collaboration with the original developers of the UP and a leading digital mental health care company with over 15 years of experience in delivering digital mental health interventions.

The UP has already shown efficacy/effectiveness in a number of trials when delivered in a face-to-face format. However, how the UP performs in an online format has yet to be examined. Online treatments have important differences with face-to-face treatments (for instance, with regard to dissemination, implementation and access to evidence-based treatments). Therefore, the findings obtained with this study could represent a very significant contribution to the literature about the UP and an Internet-delivered transdiagnostic treatment for anxiety and depression in an ecological setting in Ireland. The potential of iUP, specifically in the context of a primary care psychology service, is in its use in a stepped-care model, where iUP may show its usefulness as a low-intensity intervention suitable particularly for clients that would otherwise be on the wait-list (although the wait-list in the service where the study is going to be conducted offers some resources, it is envisaged that iUP will be superior to those resources).

Furthermore, there is literature showing that the UP leads to similar results than disorder-specific CBT [21, 22]. However, transdiagnostic treatments such as the UP offer important practical advantages compared to disorder-specific treatments, such as a better management of comorbidity. In this sense, this study would provide valuable data in a context where mental health care resources are limited. Finally, this project aligns with the e-health strategy for Ireland proposed by the Irish public health service provider, the Health Service Executive (HSE) aimed to integrate technology enabled solutions within Irish healthcare. It seeks to improve service efficiency in primary care within the HSE by providing an evidence-based solution for addressing depression, anxiety and related disorders.

If positive, the intervention tested in this project could help to increase access to empirically supported treatments, thereby reducing waiting lists to receive adequate treatment. In this way, the project will build on recent work that has deployed the SilverCloud disorder-specific interventions for anxiety and depression as part of the health services mental health care offering. While not directly studied in this project, it is likely that an intervention such as iUP, offered to people that would otherwise be on the wait-list and integrated in a stepped-care model would also show economic benefits as some studies examining similar interventions have already shown [54]. The iUP can become an important part of (public) mental health provision and can further supplement other forms of care. It can help to address the societal need for a timely and accessible provision of mental health support. Given that UP is also an intervention that is well-established in other formats such as face-to-face individual/group therapy, the availability of an internet-based version can potentially add to the layered (stepped-care) overall mental health approach, where the clients and clinician may be familiar with important intervention concepts at several levels of provision (low-intensity vs. high-intensity).

### Trials status

This is the first version of the protocol (20.5.2021), the date recruitment began (24.11.2021) and the approximate date when recruitment will be completed (31.12.2023).

## Data Availability

Once results have been published in peer-reviewed academic journals, fully anonymized data and materials will be made available on request to other researchers via the Trinity College Dublin TARA data repository, in keeping with Trinity College Dublin research policy.

## Abbreviations

CCIP: Client Change Interview Protocol
CONSORT: Consolidated Standards of Reporting Trials
DSM-5: Diagnostic and Statistical Manual of Mental Disorders, Fifth Edition
eWLC: Enhanced wait-list
GAD-7: Generalized Anxiety Disorder 7-item scale
GP: General practitioner
HSE: Health Service Executive
iCBT: Internet-based cognitive behavioural therapy
iUP: Internet-based Unified Protocol
MINI: Mini-International Neuropsychiatric Interview
OASIS: Overall Anxiety Severity And Impairment Scale
OCI-R: Obsessive-compulsive Inventory-Revised
ODSIS: Overall Depression Severity and Impairment Scale
PCL-5: Self-report measure of posttraumatic stress disorder according to DSM-5
PDSS-SR: Panic Disorder Severity Scale-Self Report
PHQ-9: Patient Health Questionnaire-9
PI: Principal investigator
RCT: Randomized controlled trial
SPIN: Social Phobia Inventory
SPIRIT: Standard Protocol Items: Recommendations for Interventional Trial
TCD: Trinity College Dublin
TMG: Trial management group
TSC: Trial steering committee
UP: Unified Protocol
WSAS: Work and Social Adjustment Scale
